# Assessment of musical interventions and its effect on blood pressure among United States populations: a systematic review and meta-analysis

**DOI:** 10.3389/fcvm.2024.1405455

**Published:** 2024-10-15

**Authors:** Shreya Meda, Joyce Gyamfi, Kahini Patel, Farha Islam, Dorice Vieira, Solomon Nyame, Christina Ruan, Krupa Boradia, Himani Chhetri, Sukruthi Thunga, Deborah Adenikinju, Etornam Amesimeku, Amy Cohen, Jumoke Opeyemi, Temitope Ojo, Carlos Chirinos, Olajide Williams, Olugbenga Ogedegbe, Emmanuel Peprah

**Affiliations:** ^1^ Department of Global and Environmental Health, ISEE Lab, NYU School of Global Public Health, New York, NY, United States; ^2^ NYU Health Sciences Library, NYU Grossman School of Medicine, New York, NY, United States; ^3^ Steinhardt School of Culture, Education, and Human Development, New York University, New York, NY, United States; ^4^ Department of Neurology, Columbia University Irving Medical Center, New York, NY, United States; ^5^ NYU Langone Medical Center, Institute for Excellence in Health Equity, New York, NY, United States

**Keywords:** hypertension, music, USA, BP reduction, musical interventions, racial/ethnic minorities

## Abstract

**Background:**

Hypertension (HTN) currently affects over 120 million Americans, in the United States (US). Thus, the implementation of evidence-based interventions (EBI) for blood pressure (BP) reduction is pivotal in minimizing this burden. We sought to evaluate evidence from published literature on the effectiveness of musical interventions for BP reduction within the US.

**Methods:**

A systematic review of studies that utilize music interventions to manage BP was conducted in October of 2022. An extensive search of several databases utilizing MeSH terms and relevant keywords was conducted for articles published through October 2022. An updated search was conducted in October 2023 to identify additional studies.

**Results:**

2,381 studies were screened for title/abstract relevancy. 1,885 studies were deemed irrelevant, and 495 studies were examined for full-text review; of which 384 were excluded due to being non-US-based. Overall, 25 studies were found where BP was the primary outcome and discussed musical interventions within the US. Of the 25 studies, 72% reported a significant decrease in BP after the administration of a musical intervention and only 28% reported the race and ethnicity of participants.

**Conclusion:**

There are limited studies that examine the effect of music interventions on BP reduction in the US, as a primary outcome. However, based on the evidence, musical interventions are effective for BP reduction. Moreover, the studies that were conducted in the US include a low percentage of high-risk racial and ethnic minority populations. Future EBI should target this underserved/high-burden group to improve disparity gaps within BP reduction via non-pharmacological means.

**Systematic Review Registration:**

Open Science Framework, doi: 10.17605/OSF.IO/4G3EB.

## Introduction

Hypertension (HTN), characterized by consistently high blood pressure (BP) is a persistent condition that burdens global populations, including racial and ethnic groups in the United States. Individuals with HTN have an increased susceptibility to cardiovascular diseases such as heart disease and stroke (1). If left untreated, HTN can lead to further complications, which requires the use of innovative tailored interventions (i.e., musical interventions) (2) as a preventative measure to curtail the impact. HTN (defined as systolic and diastolic BP ≥ 130/80) (3) was the primary or contributing cause of death for about 500,000 individuals in the United States in 2018 (1), with certain racial and ethnic minority groups being disproportionately impacted (4–6). According to current estimates, the prevalence of HTN among Black adults is highest in the United States (54%), compared to Asian adults (39%), Hispanic/Latinx adults (36%), and non-Hispanic/Latinx White individuals (46%) (1). Moreover, racial and ethnic minorities also have lower HTN control rates [Black individuals (48.5%), Hispanic/Latinx (47.4%), and Asian individuals (43.5%)] compared to non-Hispanic/Latinx White individuals (55.7%)] (7). In urban areas, the disparity in HTN prevalence and control is even greater for racial and ethnic minority groups, which may be attributed to limited access to healthcare, inadequate/poor diet, exposure to harmful pollutants/carcinogens, and other structural barriers (3, 4). Existing research has shown that Black individuals develop HTN at an earlier age than White individuals (8) due to variations in the levels of risk factors for HTN. These factors include high sodium intake, physical inactivity, unhealthy diet, (9) obesity, and stress due to racial discrimination (10, 11).

The racial disparities in HTN rates and control are growing. The disproportionate rates of HTN among various racial and ethnic groups in the United States suggest the need to implement innovative evidence-based tailored interventions for BP control among these groups. Existing evidence has confirmed that music is an effective intervention for BP control, anxiety, depression, and other physiological conditions (12–14). Therefore, a non-pharmacological intervention such as music could be beneficial for patients with high BPs. There has also been documented success of BP being controlled using music in different countries around the world (13, 15, 16).

Although music is extensively used in other parts of the world, its implementation in the United States is severely limited despite the documented advantages to lower systolic BP (SBP), diastolic BP (DBP), and heart rate (HR) (14, 17). Clinical studies have underscored the remarkable efficacy of music listening in significantly reducing SBP, DBP, HR when compared to individuals or patients who did not receive any musical intervention (13). Moreover, incorporating variable tempo and strategic pauses during music sessions has been shown to elicit favorable effects on BP, further lowering SBP, DBP, and HR (13–15). However, it is noteworthy that these studies were not conducted within the United States and had a notably limited representation of participants from diverse racial backgrounds, who could particularly benefit from such evidence-based interventions. Furthermore, these studies lacked comprehensive reporting on the specific styles of music employed, leaving a critical gap in understanding the influence of personal musical preferences and cultural relevance on health outcomes, particularly within culturally diverse communities.

Recognizing this significant gap in the existing literature, the primary objective of this review is to synthesize available evidence regarding the impact of music interventions on BP reduction within United States populations. By garnering a comprehensive understanding of this therapeutic modality, this review aims to inform evidence-based practices aimed at reducing BP and enhancing HTN control within diverse communities.

## Methods

The review protocol is registered in Open Science Framework (DOI 10.17605/OSF.IO/4G3EB).

### Search strategy

We searched PubMed/Medline, Global Health, Embase, Web of Science, Music Index, CINAHL, and Wiley Cochrane Library. We searched the grey literature in OpenGrey, WorldCat, the New York Academy of Medicine (NYAM) Grey Literature database, and references of recently published systematic reviews from inception to date. We searched PubMed Central and Google Scholar using an abbreviated strategy to ensure comprehensiveness in searching. The original search was conducted from database inception to October 2022, and updated in October 2023.

The search strategy included terms related to music, musical interventions, BP, and United States. We conducted an in-depth search using the following parameters: (music interventions OR music therapy OR community music OR musical education OR music intervention OR music medicine OR music OR musical OR singing OR songs) AND (hypertension OR high blood pressure OR blood pressure OR blood pressure determination OR diastolic OR systolic OR pulse pressure OR arterial OR pulmonary wedge OR venous OR central venous OR portal) AND (adult OR adults OR elder*OR aged) AND (Appalachian Region OR Alabama OR Georgia OR Kentucky OR Maryland OR New York OR North Carolina OR Ohio OR Pennsylvania OR South Carolina OR Tennessee OR Virginia OR West Virginia OR Great Lakes Region OR Illinois OR Indiana OR Michigan OR Minnesota OR Wisconsin OR Mid-Atlantic Region OR Delaware OR District of Columbia OR Maryland OR New Jersey OR Midwestern United States OR Iowa OR Kansas OR Kentucky OR Missouri OR Nebraska OR North Dakota OR Oklahoma OR South Dakota OR Wisconsin OR New England OR Connecticut OR Maine OR Massachusetts OR New Hampshire OR Rhode Island OR Vermont OR Idaho OR Montana OR Washington OR Wyoming OR Pacific States OR Alaska OR California OR Hawaii OR Oregon OR Alabama OR Arkansas OR Florida OR Louisiana OR Mississippi OR Arizona OR Colorado OR Nevada OR New Mexico OR Texas OR Utah OR United States OR United States OR America* OR “U.S.” OR US).

### Eligibility criteria for inclusion of studies

All citations found through the searches were downloaded to Covidence, a systematic review management tool, for title and abstract screening (18). Titles and abstracts of all articles were independently screened and rated by at least two reviewers to determine if the article meets the inclusion criteria. Following the double-screening protocol, articles were excluded if they did not contain information relevant to the systematic review objective. Articles were assessed for full-text eligibility. Eligible full-text articles were reviewed, and relevant information was extracted. The eligibility criteria included: (1) A musical intervention was used; (2) The study was conducted in the United States; (3) The study sample consisted of an Adult (18+) population; and (4) The study quantitatively measured the effect of the musical intervention on systolic and/or diastolic BP; (5) BP is a primary and or secondary outcome. Non-English studies were also included if translation was available. We excluded articles that did not meet any of the above criteria.

### Data extraction

**Figure 1:**
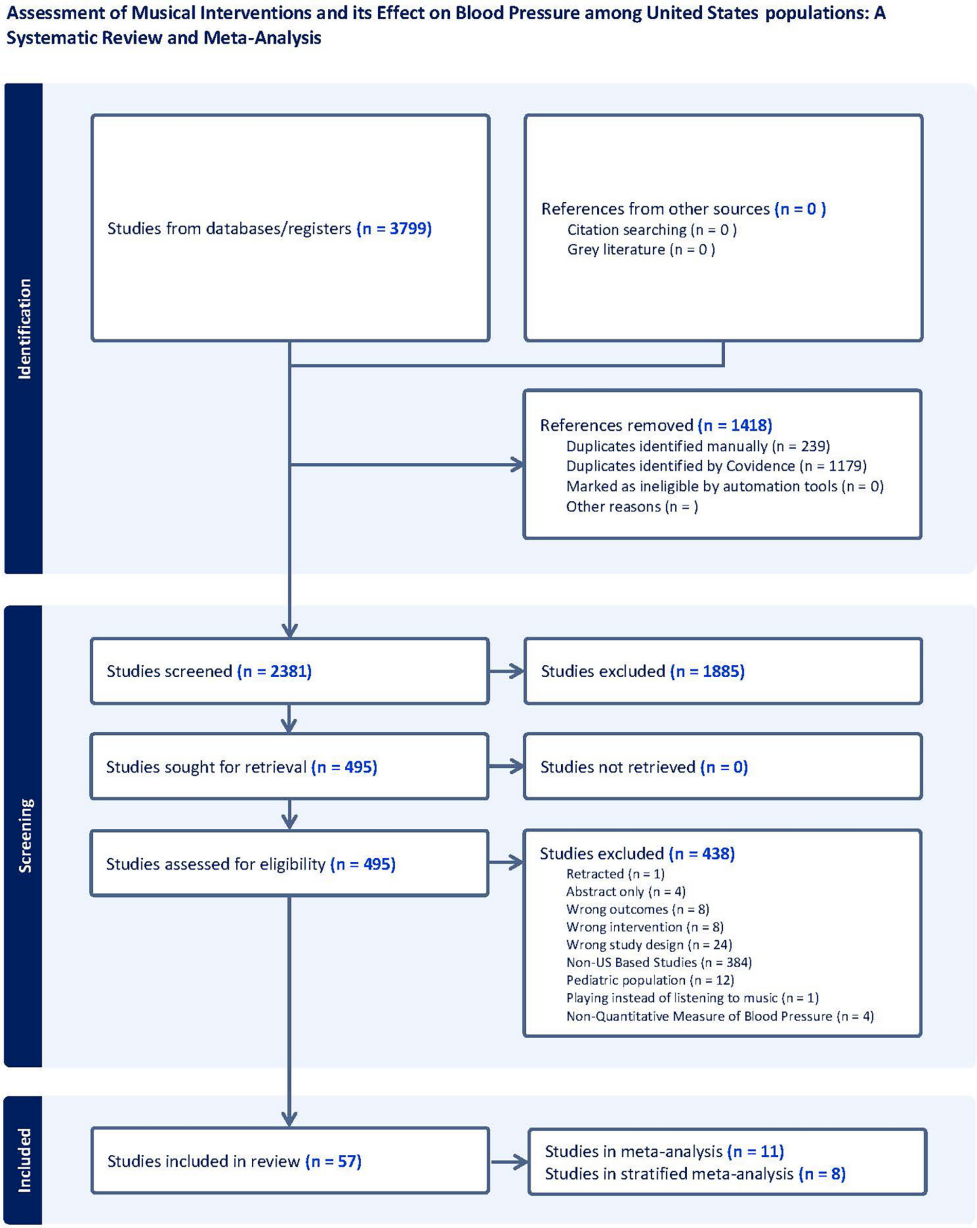
Prisma flowchart.

Full search strategies of all the databases were completed, yielding 3,799 articles in total. After duplicates were removed, 2,381 articles made it to title and abstract screening. 495 articles were included in the full-text review and examined against the review criteria. Of these, 438 articles were then excluded for various reasons including Retracted (*n* = 1), Abstract only (*n* = 4), Wrong outcome measured (*n* = 8), Wrong intervention implemented (*n* = 8), Wrong study design (*n* = 24), Non-US Based Studies (*n* = 384), Pediatric population (*n* = 12), Experiment consisted of patients playing rather than listening to music (*n* = 1), Non-numerical collection of BP (*n* = 4). 57 articles met the inclusion criteria and were examined for full-text data extraction (Figure 1).

### Quality of assessment

The risk of bias assessment was conducted using an independently designed Google Form based on the Ma et al. article that provides an assessment tool for each study design (19). Data from Covidence was extrapolated into the Google Form that incorporated the assessment for each study design. Studies that did not report study design were not included in the Risk of Bias Assessment.

### Synthesis methods and effect measure

We conducted a meta-analysis of the effect of music on BP. All meta-analyses were conducted using RevManWeb (Cochrane collaboration). A random effects model was used due to the heterogeneous nature of the studies in terms of varying study populations and non-stratification of the study outcome. Subsequently, sub-group analysis was done for the two types of studies (RCTs and non-randomized studies). Heterogeneity was assessed using Cochrane's Q and degree of inconsistency (I2). The findings of the remaining studies were presented in a narrative format.

## Results

### Characteristics of studies

Of the 57 articles, 25 articles assessed BP as a primary outcome, and the remaining 32 articles evaluated BP as a secondary outcome. Below we provide findings from the studies that examined BP as a primary outcome. Additional information for the studies that provided BP as a secondary outcome can be found in Supplementary Tables S1, S2.

A total of 25 studies reported BP as the primary outcome (20–44) (Tables 1A–2B). An additional 32 studies were included in the review that reported BP as the secondary outcome (44–74). The studies that reported BP as the primary outcome utilized RCT designs (*n* = 15), Non-RCT designs (*n* = 5), Case-Control design (*n* = 1), Mixed Methods design (*n* = 1), Qualitative design (*n* = 1), and Not Reported Study Design (*n* = 2) (Table 1A). The average sample size varied across study designs. Sample size ranged from 16 to 855 participants for RCT designs, 40–203 participants for non-RCT designs, 10–101 participants for quasi-experimental designs, 14–234 participants for other designs, and 75–254 participants for studies where the design was not reported (Table 1A). The most represented age range across all studies was middle-aged (45–64) with 19 studies including this population. Of the 25 studies, 13 studies investigated physiological responses during a music intervention. In one study, the primary purpose was to examine the autonomic response during a musical intervention, and in 10 studies, the primary purpose was to examine the response or reaction to stress/coping/anxiety/trauma during a musical intervention. The primary objective of one study was to assess the BP effects of music therapy during exercise.

**Table 1A:** Characteristics of studies assessing change in blood pressure as the primary outcome.

Author (year)	Experimental condition vs. control	Study design	Sample size	Female # (%)	Male #/(%)
Allen (20)	Tasks performed during music-free vs. self-selected vs. investigator-selection music conditions	Within subjects laboratory experiment	50	0 (0)	50 (100)
Allen (21)	Cassette with 22 types of music vs. no music during intervention	Cohort studies/non-RCT prospective & retrospective	40	30 (75)	10 (25)
Bell (22)	MP3 player preloaded with preferred instrumental classical, jazz, or rock and roll music vs. no music	Double blind	217	156 (72)	61 (28)
Bittman (23)	Gospel music program (weekly 45-min vocal and instrumental sessions) vs. no music program	RCT	71	58 (82)	13 (18)
Blanchard (24)	Rock and roll music vs. classical music vs. no music	RCT	254	130 (51)	124 (49)
Cadigan (25)	Music through a headset from a hand-held compact disc player vs. no music	Randomized, two-group, pretest/post-test design	140	40 (29)	100 (71)
Camara (26)	A classically trained pianist by study surgeon played the piano while patients were being transported into the OR from the preoperative holding area vs. no music being played	Cohort studies/non-RCT prospective & retrospective	203	137 (67)	66 (33)
Chafin (27)	Was able to chose classical, jazz, or pop music vs. no music during mental arthmetic task	Did not report	75	52 (69)	23 (31)
Cheng (28)	Classical music vs. no music	RCT	222	100 (45)	122 (55)
Geden (29)	Experiment 1 easy-listening music vs. rock music vs. self-selected music vs. dissertation (placebo-attention) Experiment 2 musical selection while using self-generated imagery vs. self-generated imagery without music vs. musical selection while using guided imagery vs. guided imagery without music vs. no music and no imagery	RCT	102	102 (100)	0 (0)
Ghetti (30)	Music therapy emotional-approach coping group vs. talk-based emotional-approach coping group vs. standard emotional-approach coping group	RCT	37	13 (35)	24 (65)
Hamel (31)	20 min of music vs. no music	Quasi experimental design	101	38 (38)	63 (62)
Maldonado-Resto (32)	Post- music therapy vs. pre-music therapy	Quasi experimental design	11	7 (63.6)	4 (36.4)
Mandel (33)	DSME/T plus MARI CD VS DSME/T plus music therapy vs. DSME/T only	Mixed methods	14	6 (43)	8 (57)
Salamon (34)	“ZZ Top” rock music vs. classical music	RCT	16	Not reported	Not reported
Schuster (35)	Dialysis plus music vs. dialysis only	Case-control	63	39 (62)	24 (38)
Sendelbach (36)	Brief session of relaxation and music via headphone for 20 min twice per day vs. 20 min of rest only	RCT	86	26 (30.2)	60 (69.8)
Smolen (37)	Subj self-selected music through headphones vs. no music	RCT	32	15 (47)	17 (53)
Steelman (38)	Choice of music vs. no music	RCT	43	Not reported	Not reported
Tang (39)	Revitalizer II program (audio guided relaxation) vs. mozart group	RCT	41	35 (85)	6 (15)
Tyndall (40)	Live preferential music (LPM) vs. no LPM	RCT	855	479 (56)	376 (44)
Vanderark (41)	Listening to music from Holst's “The Planets” for 10 min vs. sitting in silence for 10 min	Did not report	101	48 (48)	53 (52)
Volkov (42)	Music in check in area vs. no music	Qualitative study	234	145 (62)	89 (38)
Walters (43)	Music when in surgical holding area vs. no music	RCT	39	39 (100)	0 (0)
Whipple (44)	Soothing music vs. stimulating music vs. no music	Quasi experimental design	10	10 (100)	0 (0)

**Table 1B:** Characteristics of studies assessing change in blood pressure as the primary outcome.

Author (Year)	Race/Ethnicity	Age Range	Study Setting/Location
Allen (20)	Not Reported	Adult 25–44; Middle Aged 45–64	Hospital
Allen (21)	Not Reported	Middle Aged 45–64; Aged 65–79	Ambulatory surgery Center
Bell (22)	Black	Young Adult 18–24	Research Laboratory
Bittman (23)	Black	Adult 25–44; Middle Aged 45–64; Aged 65–79; Elderly 80+	Religious/Church/Temple/Mosque
Blanchard (24)	Not Reported	Young Adult 18–24; Adult 25–44	School
Cadigan (25)	Not Reported	Middle Aged 45–64; Aged 65–79	Hospital
Camara (26)	Not Reported	Middle Aged 45–64; Aged 65–79; Elderly 80+	Hospital
Chafin (27)	Not Reported	Young Adult 18–24	University
Cheng (28)	Not Reported	Middle Aged 45–64; Aged 65–79	Medical Center
Geden (29)	Not Reported	Young Adult 18–24; Adult 25–44	Laboratory
Ghetti (30)	Not Reported	Middle Aged 45–64; Aged 65–79; Elderly 80+	Hospital
Hamel (31)	Not Reported	Adult 25–44; Middle Aged 45–64; Aged 65–79	Hospital
Maldonado-Resto (32)	White; Hispanic; Mixed Race	Young Adult 18–24; Adult 25–44; Middle Aged 45–64; Aged 65–79	Clinic
Mandel (33)	Not Reported	Middle Aged 45–64; Aged 65–79	Hospital
Salamon (34)	Asian	Young Adult 18–24; Adult 25–44	School
Schuster (35)	Not Reported	Young Adult 18–24; Adult 25–44; Middle Aged 45–64; Aged 65–79; Elderly 80+	Not Reported
Sendelbach (36)	Not Reported	Young Adult 18–24; Adult 25–44; Middle Aged 45–64; Aged 65–79; Elderly 80+	Hospital
Smolen (37)	Not Reported	Young Adult 18–24; Adult 25–44; Middle Aged 45–64; Aged 65–79; Elderly 80+	Hospital
Steelman (38)	Not Reported	Young Adult 18–24; Adult 25–44; Middle Aged 45–64; Aged 65–79	Hospital
Tang (39)	White; Asian	Middle Aged 45–64; Aged 65–79; Elderly 80+	Retirement Living Facility
Tyndall (40)	Not Reported	Young Adult 18–24; Adult 25–44; Middle Aged 45–64; Aged 65–79	Emergency department
Vanderark (41)	White	Young Adult 18–24; Adult 25–44	School
Volkov (42)	Not Reported	Young Adult: 18–24; Adult: 25–44; Middle Aged: 45–64; Aged: 65–79; Elderly: 80+	Clinic
Walters (43)	Not Reported	Young Adult 18–24; Adult 25–44; Middle Aged 45–64; Aged 65–79	Hospital
Whipple (44)	White; Asian	Young Adult 18–24; Adult 25–44; Middle Aged 45–64	Laboratory

**Table 2A:** Systolic and diastolic blood pressure across studies with blood pressure as the primary outcome.

Author (year)	Sample size (T/C)	Mean SBP (mmHg) at Baseline (SD)	Mean DBP (mmHg) at Baseline (SD)	Mean SBP (mmHg) at Endpoint (SD)	Mean DBP (mmHg) at Endpoint (SD)
Allen (20)	Not reported	Not reported	Not reported	Not reported	Not reported
Allen (21)	Music: 20 Non music: 20	Music: 158 (4.2) Non music: 160 (3.8)	Music: 92 (1.4) Non music: 92 (1.4)	Music: 123 (1.5) Non music: 141 (3.6)	Music: 68 Non music: 75
Bell (22)	Classical: 56 Jazz: 56 Rock: 56 control: 55	Not reported	Not reported	Not reported	Not reported
Bittman (23)	Music: 36 No music: 35	Music: 140.99 No music: 138.48	Music: 82.88 No music: 80.33	Music: 131.26 No music: 132	Music:79.87 No music: 79.06
Blanchard (24)	No music: 82 Rock: 87 Classical: 85	No music: 118 Rock: 122 Classical: 131	No music: 58 Rock: 65 Classical: 68	No music: 147 Rock: 122 Classical: 131	No music: 108 Rock: 68 Classical: 69
Cadigan (25)	Music: 75 No music: 65	Music: 114 (18) No music: 118 (14)	Music: 60 (12) No music: 63 (14)	Music: 112 (16) No music: 121 (18)	Music: 57 (11) No music: 61 (11)
Camara (26)	Music: 115 No music: 88	Not reported	Not reported	Not reported	Not reported
Chafin (27)	Music: 75 No music: 75	Not reported	Not reported	Not reported	Not reported
Cheng (28)	Music: 112 No music: 120	Music: 141.94 (20.21) No music: 140.53 (18.80)	Music: 70.93 (11.46) No music: 64.60 (10.24)	Music: 135.72 (18.24) No music: 140.57 (20.46)	Music: 66.23 (10.24) No music: 64.99 (7.70)
Geden (29)	Not reported	Not reported	Not reported	Not reported	Not reported
Ghetti (30)	Music: 11 No music: 9	Music: 121.91 (15.99) No music: 122.00 (19.72)	Music: 63.82 (14.67) No music: 63.78 (9.35)	Music: 129.36 (17.01) No music: 128.67 (23.21)	Music: 68.55 (16.87) No music: 65.56 (16.04)
Hamel (31)	Music: 51 No music: 50	Music: 135.43 (21.82) No music: 133.44 (17.90)	Music: 73.67 (12.51) No music: 72.50 (9.44)	Music: 133.53 (19.79) No music: 139.72 (21.61)	Music: 72.78 (10.91) No music: 75.52 (11.94)
Maldonado-Resto (32)	Paired design 11	120.4 (12.4)	75.6 (7.2)	116.4 (9.5)	74.0 (7.0)
Mandel (33)	14 pre and post	119.86 (10.18)	Not reported	114.71 (9.66)	70.64 (12.28)
Salamon (34)	Classical: 8 Rock: 8	Classical: 119.875 (5.817) Rock: 109.0 (9.411)	Not reported	Classical: 112.5 (3.854) Rock: 107.875 (9.978)	Classical: 77.25 (5.418) Rock: 71.5 (6.782)
Schuster (35)	Music: 31 No music: 32	Music: 155.41	Music: 97.50	Music: 124.24	Music: 68.46
Sendelbach (36)	Music: 50 No music: 36	Music: 116 No music: 115	Music: 58 No music: 62	Music: 110 No music: 118	Music: 57 No music: 61
Smolen (37)	Music: 16 No music: 16	Music: 135 No music: 122	Music: 77 No music: 74	Music: 116 No music: 126	Music: 69 No music: 74
Steelman (38)	Music: 21 No music: 22	Music: 146.381 (22.418) No music: 139.591 (17.495)	Music: 86.047 (13.048) No music: 80.182 (7.774)	Music: 139.905 (19.491) No music: 139.864 (14.403	Music: 80.810 (11.272) No music: 81.591 (10.051)
Tang (39)	Revitalizer: 19 Mozart: 22	Revitalizer: 148 (21) Mozart: 145 (19)	Revitalizer: 78 (11) Mozart: 74 (10)	Revitalizer: 143 (17) Mozart: 139 (17)	Revitalizer: 77 (14) Mozart: 71 (10)
Tyndall (40)	Music: 432 No music: 423	Music: 139.12 (26.87) No music: 135.36 (25.07)	Music: 78.86 (16.89) No music: 78.50 (16.60)	Not reported	Not reported
Vanderark (41)	Control biology: 38 Control music: 32 Experimental biology: 14 Experimental music: 17	Control biology: 115 (10) Control music: 116 (10) Experimental biology: 123 (12) Experimental music: 121 (9)	Control biology: 77 (10) Control music: 77 (7) Experimental biology: 78 (9) Experimental music: 76 (9)	Control biology: 114 (13) Control music: 116 (10) Experimental biology: 120 (12) Experimental music: 115 (8)	Control biology: 75 (10) Control music: 77 (8) Experimental biology: 78 (10) Experimental music: 75 (9)
Volkov (42)	Music: 102 No music: 100	Not reported	Not reported	Not reported	Not reported
Walters (43)	VT: 13 M: 13 Control: 13	VT: 117.33 MT: 115.62 Control: 117.55	VT: 66.73 MT: 60.44 Control: 65.77	VT: 124.03 MT: 133.54 Control: 126.66	VT: 74.67 MT: 81.38 Control: 76.38
Whipple (44)	Total: 10	Not reported	Not reported	Not reported	Not reported

**Table 2B:** Mean blood pressure difference across studies with blood pressure as the primary outcome.

Author (Year)	Mean SBP (mmHg) Difference (SD)	Mean DBP (mmHg) Difference (SD)	Was Difference Statistically Significant?	Were patients with HTN Included?
Allen (20)	Not Reported	Not Reported	Yes	Not Reported
Allen (21)	Not Reported	Not Reported	Yes	No
Bell (22)	Not Reported	Not Reported	Yes	Not Reported
Bittman (23)	Not Reported	Not Reported	Yes SBP (In both groups when looking at baseline BP)	Yes
Blanchard (24)	Not Reported	Not Reported	Yes	Not Reported
Cadigan (25)	Not Reported	Not Reported	Yes	Not Reported
Camara (26)	Not Reported	Not Reported	Yes	Not Reported
Chafin (27)	Not Reported	Not Reported	Yes	Not Reported
Cheng (28)	Music: 6.21 (9.83) No music: −0.05 (11.24)	Music: 4.70 (8.88) No music: −0.39 (5.75)	Yes	Not Reported
Geden (29)	Not reported	Not reported	Not Reported	Not Reported
Ghetti (30)	Not Reported	Not Reported	Yes	Not Reported
Hamel (31)	Not Reported	Not Reported	Yes	Not Reported
Maldonado-Resto (32)	−4	−1.6	No	Yes
Mandel (33)	Not Reported	Not Reported	Yes	Not Reported
Salamon (34)	Not Reported	Not Reported	Yes	Not Reported
Schuster (35)	Not Reported	Not Reported	No	Not Reported
Sendelbach (36)	Not Reported	Not Reported	No	Not Reported
Smolen (37)	Not Reported	Not Reported	Yes	Not Reported
Steelman (38)	Not Reported	Not Reported	Yes	Not Reported
Tang (39)	Not Reported	Not Reported	Yes	Not Reported
Tyndall (40)	Music: 9.03	Music: 5.86	Yes	Not Reported
Vanderark (41)	Not Reported	Not Reported	No	Not Reported
Volkov (42)	Music: −29.26 No Music: −11.17	Music: −5.99 No Music: −3.98	Yes	Yes
Walters (43)	Vibrotactile Therapy: 6.7 Music Therapy: 17.92 Control: 9.11	VT: 7.94 MT: 20.94 Control: 10.61	No	Not Reported
Whipple (44)	Not Reported	Not Reported	No	Not Reported

### Intervention administration for studies reporting BP as a primary outcomes

Intervention durations differed across all 25 studies. Duration of musical interventions ranged from 10 min to 2.5 h; however, the number of times the intervention was administered on an hourly, weekly, or monthly basis varied across the studies. Only five studies reported administering the intervention twice or more. Among these five studies, there are variations in the period of the study duration with the range being 1 day to 1 year. Aside from these five studies, 14 studies reported providing the musical intervention once, and six studies did not report or were unclear on the duration of the musical interventions.

Overall, interventions were conducted in hospitals (*n* = 10), clinics (*n* = 2), schools (*n* = 3), laboratories (*n* = 3), a religious center (*n* = 1), a retirement living facility (*n* = 1), an emergency department (*n* = 1), an ambulatory surgery center (*n* = 1), and a medical center (*n* = 1). Several of the studies reviewed included a sample of patients undergoing medical procedures including Camara et al. (26), Cheng et al. (28), Hamel et al. (31), Schuster et al. (35), Sendelbach et al. (36), Smolen et al. (37), Steelman et al. (38), and Walters et al. (43) The musical interventions were done in the preoperative period (*n* = 3), during the procedure (*n* = 3), and in the postoperative period (*n* = 2), with the intervention in Allen et al. (21) administered during the preoperative, surgical, and postoperative periods. Mandel et al. (33), Tyndall et al. (40), and Volkov et al. (42) administered the musical intervention to a patient population not undergoing procedures. Blanchard et al. (24), Salamon et al. (34), and Vanderark et al. (41) utilized a student population. Whipple et al. (44) used a college graduate sample.

Listening to recorded music was an essential part of the interventions administered in all the studies reviewed. Some studies did have additional components in the intervention. Allen et al. (20) and Chafin et al. (27) asked participants to do a mental arithmetic task during the music listening. Bittman et al. (23) enrolled participants into a gospel music program that had both vocal and instrumental sessions. Blanchard et al. (24) instructed students to take a final examination during the music listening. Ghetti et al. (30) incorporated emotional support coaching into the intervention. Maldonado-Resto et al. (32) asked participants to exercise. Finally, Geden et al. (29) used guided imagery along with the music listening.

### Ethnicity of participants in which BP was a primary outcome

Of the 25 studies, only 7 (28%) of studies reported the race and ethnicity of participants. 18 studies (72%) did not report the ethnic or racial background of participants. Of the studies that reported race and ethnicity, Bell et al. (22) and Bittman et al. (23) included only Black participants. Vanderark et al. (41) included only white participants. Salamon et al. (34) included solely Asian participants. Whipple et al. (44) and Tang et al. (39) included White and Asian participants. Maldonado-Resto et al. (32) included White and Hispanic/Latinx Participants. Overall, among the 7 studies that reported the race and ethnicity of participants, 4 Studies had White Participants, 3 Studies had Asian Participants, 2 Studies had Black Participants, and 1 Study had Hispanic/Latinx Participant.

### Impact of diverse music genres on BP

Among the 25 studies, a diverse array of musical genres was employed as part of the interventions. These included Classical Music, Rock and Roll Music, Jazz Music, and live piano performances, among others. Notably, 8 studies assessed the effectiveness of classical music on reducing BP. Within these studies, 7 of them reported statistically significant reduction of BP measurements.

Several studies individually evaluated the efficacy of each music genre in reducing BP. For example, a study that included Classical music with a robust sample size was conducted by Bell and colleagues (22). In this study, the experimental group of 162 participants listened to music for 30 min from 1 of the 3 genres offered (Classical, Classic jazz, or Classic rock and roll). The intervention was administered 4 times per week for a total of 12 weeks. The control condition which consisted of 55 participants was instructed to not listen to any music and to sit at the table quietly for 30 min during the entire study duration. Following the intervention, participants measured their BP using an ambulatory BP machine and noted their post-intervention BP. The change in SBP in the experimental condition was statistically significant at 14.26 mmHg lower.

### Live vs. recorded music on blood pressure reduction

Within the studies where BP was the primary outcome that was measured, several studies utilized live music as the medium for musical intervention compared to recorded music. Out of the 25 studies, 2 studies reported using live music as part of the musical intervention. Camara (26) measured the effect live piano music had on patients when they were being transported from the preoperative holding area to the operating room. Compared to the control group, it was found that the patients that listened to live piano music during their transport had BP measurements in the Operating Room that was significantly lower than what was measured in the pre-operating room. In Tyndall (40), patients who were in the emergency room were subjected to Live Preferential Music in the form of live guitar or live vocal music. When compared to patients who did not receive Live Preferential Music during their time in the Emergency Room, the patients who did receive the live music had statistically significant BP reductions, which was not seen in the control group.

### Quantitative analysis of BP outcomes in music intervention studies

Tables 2A, 2B displays the quantitative SBP/DBP values reported by the studies where BP was the primary outcome. Among the 25 studies that reported BP as the primary outcome, the mean baseline SBP/DBP, endpoint SBP/DBP, and difference in SBP/DBP were not reported in six studies. The mean difference in SBP/DBP was not reported in seventeen studies (68%). Out of the 25 studies, 18 reported statistically significant differences (72%) in SBP and DBP measurements for participants in the intervention arm. When measured, BP was collected by a manual sphygmomanometer or by an automated BP monitor.

### BP as a secondary outcome measure in music intervention

An additional 32 studies were included in the review that reported BP as the secondary outcome (45–75). These studies aimed to investigate the use of musical interventions on anxiety, psychological distress, pain, relaxation, and satisfaction. Anxiety was the primary outcome that was measured for most of the studies, and pain was a primary outcome for a few studies. Nevertheless, across the studies, BP was assessed as a secondary variable to gauge the effects on the primary outcome. Interestingly, the analysis revealed a significant reduction in BP in 10 out of the 32 studies (31%). Additional details for these studies can be found in Supplementary Tables S1, S2.

### Risk of bias assessment

**Figure 2:**
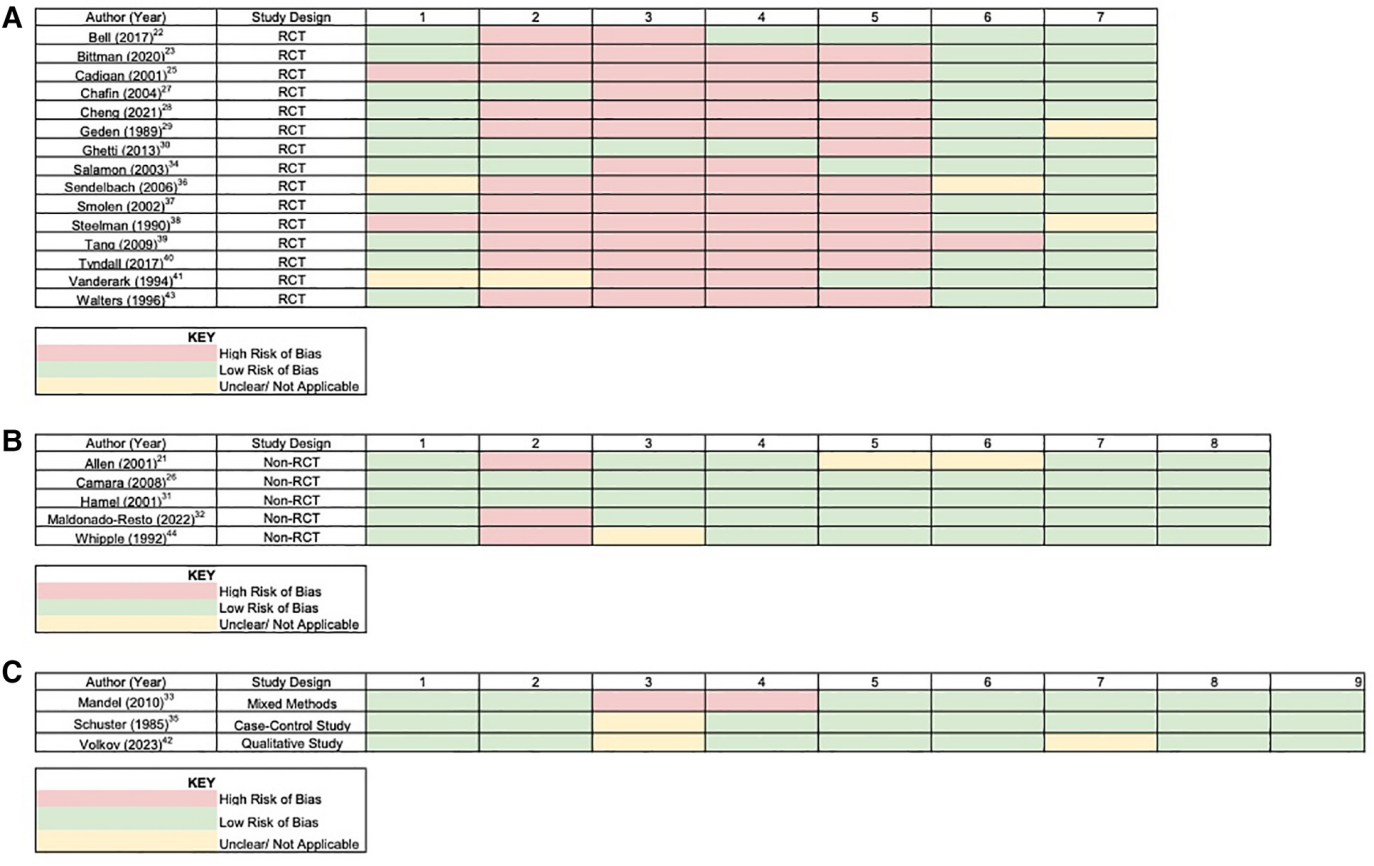
**(A)** Risk of bias table—RCT studies. Column numbers represent the criteria of the risk of bias assessment for randomized control trials: (1) random sequence generation; (2) allocation concealment; (3) blinding of participants; (4) blinding of personnel; (5) blinding of outcome assessors; (6) incomplete outcome data; (7) selective reporting. **(B)** Risk of bias table—non-RCT studies. Column numbers represent the criteria of the risk of bias assessment for non-randomized control trials: (1) clarity of cause and effect; (2) participants receiving in similar treatments other than intervention; (3) control group; (4) several measurements pre and post intervention; (5) complete follow-up; (6) outcomes compared similarly; (7) outcomes measured reliably; (8) appropriate statistical analysis. **(C)** Risk of bias table—other studies. Column numbers represent the criteria of the risk of bias assessment for non-randomized control trials: (1) clarity of cause and effect; (2) participants compared similarly; (3) participants receiving in similar treatments other than intervention; (4) control Group; (5) several measurements pre and post intervention; (6) complete Follow-up; (7) outcomes compared similarly; (8) outcomes measured reliably; (9) appropriate statistical analysis.

Overall, based on the results in Figure 2, there is a low risk of bias within the results that were reported by the studies where BP was a primary outcome of measurement. One major source of bias that may have affected the reporting of the outcomes is that most of the studies using the RCT design did not have allocation concealment, blinding of participants, or blinding of personnel. Consequently, this limits the anonymity of the participants to the research staff conducting the final physiological measurements. However, due to the inherent nature of the studies and the impracticality of implementing a placebo, achieving true blinding and allocation concealment may have been challenging if not impossible.

### Meta-analysis findings

**Figure 3:**
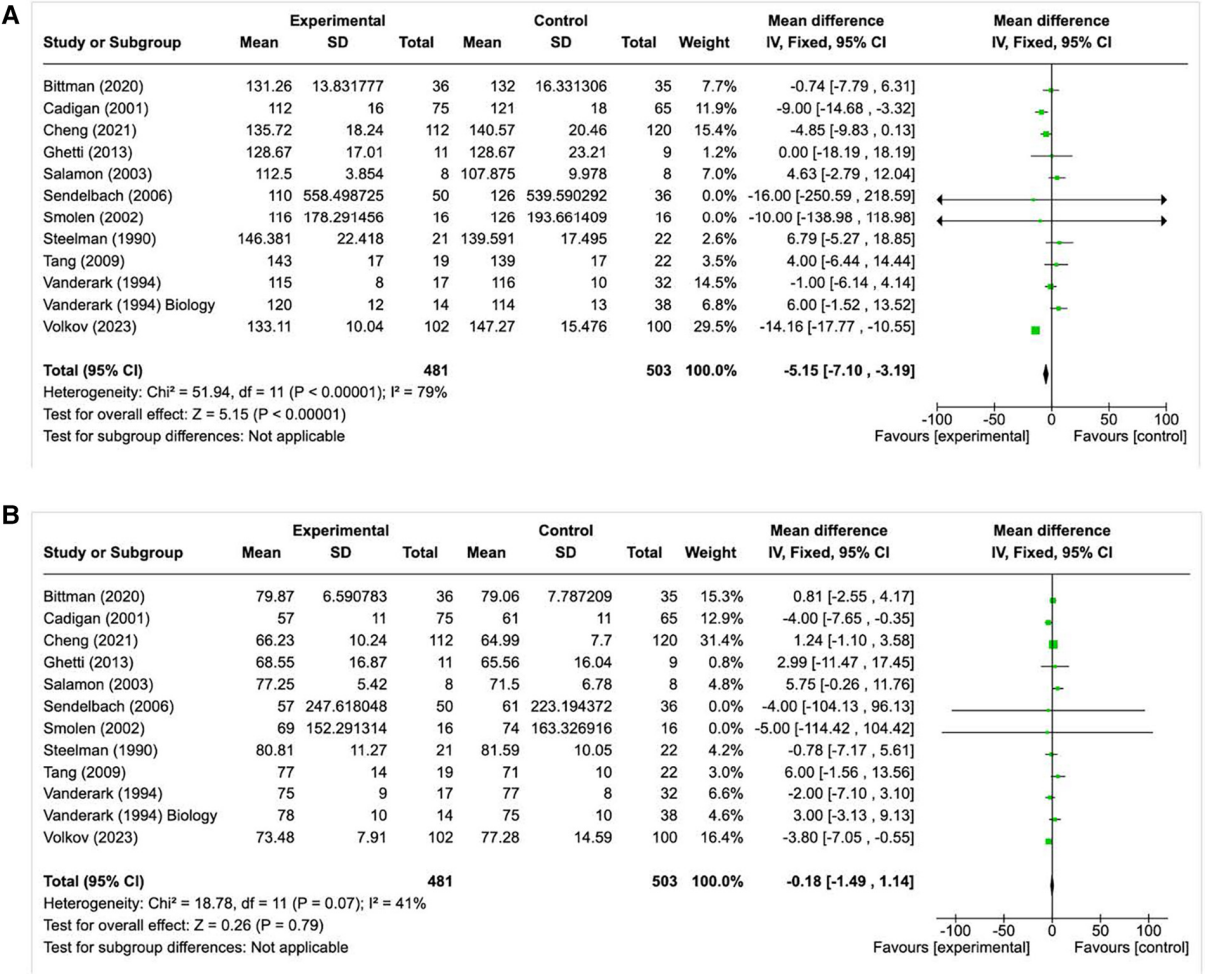
**(A)** Forest plot for studies highlighting the effect of music on systolic BP (BP as a primary outcome). **(B)** Forest plot for studies highlighting the effect of music on diastolic BP (BP as a primary outcome).

Among the 25 studies examining BP as a primary outcome, those that reported the effect of music intervention on systolic and diastolic BP, and provided sufficient data were included in the meta-analysis. The pooled results from eleven studies, with one study being split into two due to different participant arms, (*n *= 984; Experimental = 481 and Control = 503) reporting on the effect of music intervention regarding systolic BP were found to favor the music intervention. The results showed the pooled Mean Difference as −5.15 mmHg (95% CI −7.10 to −3.19) with greater evidence of heterogeneity between these studies (I2 = 79%, *p*-value = 0.00001). In contrast to these findings, the pooled results from these eleven studies reporting on the effect of music intervention for diastolic BP did not favor the music intervention. The results showed minimal reduction in DBP (Pooled Mean Difference −0.18 mmHg; 95% CI −1.49 to −1.14) with little evidence of heterogeneity between the studies (I2 = 41%, *p*-value = 0.79) (Figures 3A,B).

## Discussion

Our investigation delved into the efficacy of musical interventions in lowering BP among adults in the United States. In studies where BP served as the primary outcome measurement, compelling evidence emerged, indicating a statistically significant reduction in BP through musical interventions. Among the 25 studies prioritizing BP as the primary outcome, a unique approach involved employing a single-encounter musical intervention to assess the impact of music on BP levels preceding either a medical procedure or an office visit (Table 1A). There were very few studies that examined the effect that musical interventions had on BP for a longer duration of time. Among the studies, the intervention duration ranged from 10 min to two and half hours. The findings of this systematic review underscore the necessity for additional research focusing on evidence-based musical interventions, specifically targeting BP as a primary outcome. In addition to this, 3 out of the 25 studies (12%) where BP was the primary outcome (Table 2B) reported that patients with HTN diagnosis were included in the sample. There is clear evidence of limited information on how musical interventions can help reduce BP in the United States. Such studies should also be extended over a longer duration, instead of a single encounter, to effectively ascertain their potential as non-pharmacological solutions for BP reduction and HTN control.

Within this systematic review, 18 of the studies neglected to report the racial or ethnic composition of their participants. Among the seven studies that did provide this information, there was a notable disparity: four studies predominantly included White participants, three studies had mostly Asian participants, two studies focused on Black participants, and one study centered on Hispanic/Latinx participants. The existing evidence suggests a serious gap in the reporting of demographic information for study participants. Additionally, fewer studies examined interventions for Black and Hispanic/Latinx communities, two ethnic groups that are disproportionately burdened by HTN in the United States. There is a clear paucity of studies that report racial and ethnic makeup. This stark underrepresentation of racial and ethnic minorities in the primary literature underscores the urgent need for future studies to encompass a more diverse sample to accurately assess the impact of music interventions on BP reduction among various racial and ethnic minority populations.

Furthermore, among the studies that included mixed racial and ethnic minority populations, three studies failed to stratify their results and report them based on population demographics. This oversight has significant implications, as it hinders our ability to discern any population-specific effects within the interventions studied. Given that high BP and related disorders disproportionately affect racial and ethnic minority populations, particularly Black and Hispanic/Latinx communities compared to Caucasian communities, it is imperative that medical interventions and policy decisions adequately account for and address these disparities.

To further examine how music can help reduce BP, specifically in Black and Hispanic/Latinx communities, tailored cost-effective, and feasible interventional studies are needed. This will allow for high BP to be addressed among this group, leading to long-term systematic changes to BP treatment protocol within the healthcare system. This will eventually allow healthcare systems to avoid multi-system downstream effects of uncontrolled high BP/HTN (e.g., stroke, kidney disease, etc.).

Based on the studies conducted where BP was the primary outcome, only two studies looked at the effect of live music on reducing BP. Within these studies, it was found that BP was reduced significantly in the intervention group compared to the control group which comprised of participants who did not receive the live music intervention. However, due to the limited number of studies that only looked at live music, we cannot draw conclusions about the overall effectiveness of live music vs. recorded music on reducing BP. In addition to this, the studies that used live music for their musical intervention had very short intervention durations, lasting at most 2 h. Based on this, it is evident that there is a need for studies to be conducted that encompass a longer intervention duration to evaluate the true potential of live music for BP reduction.

Music was also shown to be an effective non-pharmacological therapy when implemented over an extended duration. For the Bell et al. study (22), participants completed the musical intervention for 30 min each week over 12 weeks. In the Bittman et al. (23) study, the participants completed the musical intervention for 45 min every other week for a total duration of a year. The researchers found that the positive healthcare outcomes were due to participants being exposed to the repetitiveness and abundance of musical interventions. Therefore, by increasing the time to which music is listened to, a greater reduction in BP may be achieved. This finding is in alignment with evidence from a recent systematic review showing that multi-component interventions over a longer duration of time yield statistically significant decreases in BP (76). Intervention duration or music exposure dosage, and music type should be considered along with other proven therapies when designing evidence-based interventions to reduce BP among racial and ethnic minorities at the population level.

Among studies conducted within the United States, it has been shown that classical music reduced BP greater than other types of music. Bell et al. (22) was able to compare the different types of music on overall BP reduction. The authors concluded that music interventions were indeed effective in reducing BP. Moreover, the findings indicate that classical music notably outperformed other genres such as jazz and rock and roll in significantly reducing participants’ BP levels. This suggests a distinct advantage of classical music over jazz and rock and roll in its impact on BP reduction. Additionally, a study conducted by Chafin et al. (27) measured how various types of music can affect BP during a stressful arithmetic session. In comparison to pop music, jazz music, and no music; classical music was able to significantly reduce BP by 2.1 mmHg after the stressful task when compared to the baseline. This study shows that even within stressful situations, classical music still has the potential to significantly reduce BP compared to other types of music. In addition to this, it has been found that relaxing musical stimuli causes an increase in cardiac vagal modulation and cardiac vagal tone, which is not affected by other types of music (77). The findings from Bell et al. (22) and Chafin et al. (27) emphasize the therapeutic potential of a non-pharmacological intervention including classical music to help reduce BP due to the effect classical and relaxing music has on physiological parameters.

It is crucial to underscore the familiarity and effectiveness of utilizing music interventions for HTN control, as evidenced by successful implementation internationally. For example, researchers in Turkey (17) investigated the effects of Turkish classical music on HTN patients revealing significant positive effects. Notably, the study demonstrated a mean reduction of SBP of 13.00 mmHg in the experimental group, and 6.50 mmHg in the control group (17).

Similarly, a study in Brazil investigated the efficacy of a musical intervention in alleviating anxiety and improving vital parameters among individuals with head and neck cancer. The experimental group, exposed to a classical piece called “Spring” from The Four Seasons by Vivaldi (15) demonstrated remarkable reductions in BP, with a 95% decrease in systolic BP and a 55% decrease in diastolic BP, compared to the control group's reductions of 55% and 30%, respectively. Furthermore, a study conducted with hemodialysis patients in Italy found that live saxophone music therapy significantly reduced BP and enhanced quality of life, with statistical significance (*p* < .05) (78). These studies combined with the US-based studies that looked at classical music collectively accentuate the effectiveness of classical and culturally relevant musical interventions in reducing BP. Moreover, these studies shed light into possible musical interventions beneficial for racial and ethnic minority populations, offering promising avenues for long-term HTN management.

Based on our findings, studies conducted in the United States on music interventions and BP reduction specifically with racial and ethnic minority populations remains scarce. However, given the success of music interventions in other countries, there is an opportunity to replicate such interventions tailored specifically for high-risk racial and ethnic minority populations in the United States. For instance, a notable US-based study targeted stroke prevention, an outcome often associated with uncontrolled HTN, through a culturally tailored musical intervention, has had great success. William and colleagues introduced “Hip-Hop Stroke,” a program in central Harlem, New York City, designed to enhance stroke knowledge using culturally and age-appropriate music and dance alongside an interactive didactic stroke curriculum incorporating the FAST mnemonic (Facial droop, Arm weakness, Speech disturbance, Time to call 911). (79) The findings highlighted the effectiveness of this approach, as students retained stroke knowledge due to the cultural relevance of the music, particularly in a community with high stroke risk. Although this study did not investigate how music, as itself, helps to reduce BP, it highlights how music exposure is very feasible for a large population, allowing for future EBIs to follow a similar protocol.

Moving forward, it is imperative for future studies to focus on culturally appropriate musical interventions to control HTN for racial and ethnic minority populations in the United States and abroad, akin to the Hip-Hop stroke project. Exploring the role of cultural appropriateness of music in facilitating acceptance, uptake, and adherence to music interventions for BP management in culturally diverse communities holds promise for advancing HTN control efforts.

## Conclusion

The findings derived from this systematic review strongly indicate the effectiveness of musical interventions in reducing BP among at-risk populations in the United States, particularly through the utilization of classical music either independently or in conjunction with other musical genres. However, a significant deficiency exists in the reporting of racial and ethnic data, a critical aspect necessary for informing culturally tailored interventions aimed at enhancing the acceptability, uptake, sustainability, and scalability of interventions. Addressing this gap is crucial for advancing health equity in HTN management.

Moreover, there is an urgent need for improvements in data reporting and transparency within research studies. Additionally, further research is warranted to thoroughly investigate the benefits of culturally appropriate musical interventions for BP reduction among racial and ethnic minority populations. Such endeavors are essential for optimizing HTN management strategies and fostering health equity across diverse communities.

## Data availability statement

The original contributions presented in the study are included in the article/Supplementary Material, further inquiries can be directed to the corresponding author.
